# Human Plasmablast Migration Toward CXCL12 Requires Glucose Oxidation by Enhanced Pyruvate Dehydrogenase Activity *via* AKT

**DOI:** 10.3389/fimmu.2018.01742

**Published:** 2018-07-27

**Authors:** Hyo-Kyung Pak, Bora Nam, Yoon Kyoung Lee, Yong-Woo Kim, Jin Roh, Jaekyoung Son, Yoo-Sam Chung, Jongseon Choe, Chan-Sik Park

**Affiliations:** ^1^Department of Pathology, Asan Medical Center, University of Ulsan College of Medicine, Seoul, South Korea; ^2^Institute for Life Sciences, Asan Medical Center, University of Ulsan College of Medicine, Seoul, South Korea; ^3^Department of Pathology, Ajou University School of Medicine, Suwon, South Korea; ^4^Department of Biomedical Sciences, University of Ulsan College of Medicine, Seoul, South Korea; ^5^Department of Otolaryngology, Asan Medical Center, University of Ulsan College of Medicine, Seoul, South Korea; ^6^Department of Microbiology and Immunology, School of Medicine, Kangwon National University, Chuncheon, South Korea

**Keywords:** CXCL12, plasmablast, AKT, glucose oxidation, pyruvate dehydrogenase, ATP, myosin light chain, humoral immunity

## Abstract

Migration of human plasmablast to the bone marrow is essential for the final differentiation of plasma cells and maintenance of effective humoral immunity. This migration is controlled by CXCL12/CXCR4-mediated activation of the protein kinase AKT. Herein, we show that the CXCL12-induced migration of human plasmablasts is dependent on glucose oxidation. Glucose depletion markedly inhibited plasmablast migration by 67%, and the glucose analog 2-deoxyglucose (2-DG) reduced the migration by 53%; conversely, glutamine depletion did not reduce the migration. CXCL12 boosted the oxygen consumption rate (OCR), and 2-DG treatment significantly reduced the levels of all measured tricarboxylic acid (TCA) cycle intermediates. AKT inhibitors blocked the CXCL12-mediated increase of OCR. CXCL12 enhanced the pyruvate dehydrogenase (PDH) activity by 13.5-fold in an AKT-dependent manner to promote mitochondrial oxidative phosphorylation. The knockdown and inhibition of PDH confirmed its indispensable role in CXCL12-induced migration. Cellular ATP levels fell by 91% upon exposure to 2-DG, and the mitochondrial ATP synthase inhibitor oligomycin inhibited CXCL12-induced migration by 85%. Low ATP levels inhibited the CXCL12-induced activation of AKT and phosphorylation of myosin light chains by 42%, which are required for cell migration. Thus, we have identified a mechanism that controls glucose oxidation *via* AKT signaling and PDH activation, which supports the migration of plasmablasts. This mechanism can provide insights into the proper development of long-lived plasma cells and is, therefore, essential for optimal humoral immunity. To our knowledge, this study is the first to investigate metabolic mechanisms underlying human plasmablast migration toward CXCL12.

## Introduction

Humoral immunity plays a pivotal role in protecting the body by producing antibodies (Abs) specific to invading pathogens (1, 2). The fundamental factors required for successful Ab production are the generation and maintenance of long-lived plasma cells. The migration of plasmablast to the bone marrow niche is prerequisite for the proper development of long-lived plasma cells (3, 4). The deletion of the chemotactic receptor CXCR4, which is required for the migration to the bone marrow triggered by the CXCL12 homing signal, decreases the number of Ab-secreting cells (5), serum immunoglobulin (Ig) levels, and the number of plasma cells in the bone marrow (6). Therefore, the migration of plasmablasts to the bone marrow niche is essential for optimal humoral immunity.

Plasma cells are one of the most intriguing cells in terms of the metabolic function because they produce up to 2,000 Ig molecules per second and form 10^5^ disulfide bonds per second to support Ig synthesis (7, 8). Moreover, the lifespan of plasma cells is estimated to decades in the hypoxic bone marrow (9). Recent studies have made some progress with elucidating the peculiar metabolism of plasma cells. Glucose is essential for the glycosylation of Igs and for the maintenance of long-lived plasma cells (10). Glutamine is crucial in Ig secretion by plasma cells (11). In addition, fatty acids play an important role in endoplasmic reticulum expansion during plasma cell differentiation (12). However, to our knowledge, no report has examined the metabolic pathways responsible for the migration of plasma cells toward the bone marrow niche (13–18).

AKT signaling is a major pathway involved in blood cell migration. AKT phosphorylation promotes neutrophil invasion, and the inhibition of AKT activity blocks its migration (19). PI3K-AKT signaling controls CD8^+^ T cell migration (20). In addition, AKT increases mesoderm cell migration (21). In addition, endothelial cell migration is increased by AKT signaling (22). Consistent with these reports, we had previously demonstrated that the migration of human plasmablasts toward CXCL12 is dependent on AKT activation (23). Moreover, AKT is a key regulator of cellular metabolism (24) and plays a central role in promoting glucose metabolism in activated T cells by increasing glucose uptake and hexokinase activity (25). IGF1-induced AKT phosphorylates pyruvate kinase M2 and regulates its activity (26). However, the metabolic role of AKT in plasmablast migration has not been investigated.

Here, we investigated the major metabolic pathways underlying human plasmablast migration. We show for the first time that the migration of plasmablasts in response to CXCL12 is highly dependent on glucose and that CXCL12-induced AKT activation increases pyruvate dehydrogenase (PDH) activity to increase glucose oxidation. The results suggest a specific mechanism underlying glucose use by plasmablasts migrating toward CXCL12; this mechanism is crucial for proper development of long-lived plasma cells and is, therefore, essential for optimal humoral immunity.

## Materials and Methods

### Reagents and Antibodies

Glucose solution (A24094001) and l-glutamine (25030081) were purchased from Thermo-Fisher Scientific (Waltham, MA, USA). Methyl-pyruvate (371173), 2-DG (D8375), 6-diazo-5-oxo-L-norleucine (DON; D2141), oligomycin A (75351), rotenone (R8875), antimycin A (A8674), and carbonyl cyanide-4-phenylhydrazone (FCCP; C2920) were obtained from Sigma-Aldrich (St. Louis, MO, USA). AMD 3100 (S8030), GSK690693 (S1113), and MK-2206 (S1078) were purchased from Selleckchem (Houston, TX, USA). Recombinant human CXCL12 (300-28A) and interleukine (IL)-21 (200-21) were purchased from PeproTech (Rocky Hill, NJ, USA). IL-2 was obtained from Hoffmann-La Roche (Basel, Switzerland). Ficoll-Paque PLUS was purchased from GE Healthcare (Marlborough, MA, USA). Human reference serum (RS10-110) was obtained from Bethyl (Montgomery, TX, USA). TMB substrate (34021), SuperSignal West Pico Chemiluminescent Substrate (34080), and 4′,6-diamidino-2-phenylindole dihydrochloride (DAPI; D1306) were purchased from Thermo-Fisher Scientific.

FITC mouse IgG1 isotype control (553971), FITC mouse anti-human TCRα/β (561673), FITC mouse anti-human CD138 (552723), PE mouse IgG1 isotype control (553972), PE mouse anti-human CD20 (555623), PE mouse anti-Akt (pS473; 560378), PerCP-Cy5.5 mouse IgG1 isotype control (555751), and APC mouse anti-human CXCR4 (560936) were obtained from BD Biosciences (Franklin lakes, NJ, USA). FITC mouse anti-human Ki-67 (11-5699) was purchased from Thermo-Fisher Scientific, and FITC-conjugated anti-rabbit secondary Ab (111-096-003) was obtained from Jackson ImmunoResearch Laboratories (West Grove, PA, USA). Goat anti-human Ig(H + L)-UNLB (2010-21) and goat anti-human Ig(H + L)-HRP were purchased from Southern Biotech (Birmingham, AL, USA). Phospho-PDH-E1α subunit (Ser293; Ab92696) was obtained from Abcam (Cambridge, UK). PDH (C54G1) rabbit mAb (3025), anti-rabbit IgG, HRP-linked antibody (7074), anti-mouse IgG, HRP-linked antibody (7076), anti-human myosin light chain (MLC) 2 antibody (3672), and anti-human phospho-MLC 2 (Ser19) mouse mAb (3675) were purchased from Cell Signaling Technologies (Danvers, MA, USA). Anti-human β-actin antibody (C4; Sc-47778) was obtained from Santa Cruz Biotechnology (Dallas, TX, USA). Miltenyi goat anti-mouse magnetic microbeads (130-048-402) were purchased from Miltenyi Biotec (Bergisch Gladbach, Germany).

### *In Vitro* Generation of Migrating Plasmablasts

Human tonsils were obtained from the remaining tissues after a routine tonsillectomy and handled in accordance with an IRB-approved protocol (2013–0864). Tonsillar mononuclear cells (MNCs) were extracted by mechanical disruption. Briefly, specimens were cut into fragments (3–10 mm), placed in RPMI 1640 containing 10% bovine calf serum (BCS), and mashed using scissors and forceps. The extracted cells were then collected, and tissue debris was removed using a disposable pipette. The cell suspension was overlaid on Ficoll-Paque PLUS, and then, Ficoll density gradient centrifugation was performed. The lymphocyte layer (interface layer containing MNCs) was then collected.

Germinal center-B (GC-B) cells were purified from tonsillar MNCs using magnetic-activated cell sorting (MACS). Briefly, 3 × 10^7^ MNCs were incubated with mouse anti-IgD, mouse anti-CD3 (OKT3), and mouse anti-CD44 in phosphate-buffered saline (PBS) for 20 min in the dark on ice. After washing with RPMI 1640 containing 10% BCS, the cells were incubated with goat anti-mouse magnetic microbeads according to the manufacturer’s instructions. The cells were then washed and resuspended in RPMI 1640 containing 10% fetal bovine serum. The LS magnetic separation column (130-042-401; Miltenyi Biotec) was placed in a magnet, and then, the cell suspension was applied to the top of the column and allowed to pass through; the effluent was collected as the negative fraction. Cell purity was greater than 95% as assessed by CD20 and CD38 expression.

To generate migrating plasmablasts, CD40L-expressing mouse L cells (2 × 10^4^ cells/mL) or HS-5 human stromal cells (1 × 10^5^ cells/mL) were irradiated with 5,000 rad and seeded onto a 24-well plate 1 day before adding GC-B cells. The *in vitro* GC-B cell differentiation to plasmablast was performed *via* two step cultures because the presence of CD40L in initial GC-B cells culture is essential for the survival of GC-B cells *per se* whereas, CD40L can inhibit the differentiation of GC-B cells to plasmablasts. First, isolated GC-B cells (2 × 10^5^ cells/mL) were cultured with irradiated CD40L-expressing L cells in presence of interleukin (IL)-2 (30 U/mL) and IL-21 (30 ng/mL) for 4 days. Subsequently, the cultured cells were harvested, and the 1 × 10^5^ cells were secondly cultured with irradiated HS-5 human stromal cells in presence of IL-2 (30 U/mL) and IL-21 (30 ng/mL) for 3 days. Differentiation was assessed according to the expression levels of *Bcl-6* and *Blimp*-*1* (both measured by qPCR) as well as CD38 and CD20 (both measured by flow cytometry). Migration was analyzed using a transwell migration assay.

### Flow Cytometry

Plasmablasts were incubated on ice for 20 min with antibodies in the flow cytometry buffer [PBS containing 1% bovine serum albumin (BSA)]. Next, the cells were washed three times with the flow cytometry buffer, and then, 20,000 events per sample were acquired using an Accuri C6 flow cytometer (BD Biosciences). Data were analyzed using FlowJo software (FlowJo LLC, Ashland, OR, USA).

### Quantitative PCR

Total cellular RNA was purified using NucleoSpin RNA (740955, Macherey-Nagel, Düren, Germany). cDNA was synthesized from 1 µg of total RNA using the iScript cDNA Synthesis Kit (Bio-Rad Laboratories, Hercules, CA, USA). The following gene-specific primers were used: Blimp-1, 5′-ATCTCAGGGCATGAACAAGG-3′ (sense) and 5′-ATGGGAAGGCTATGCAAACA-′ (anti-sense); Bcl-6, 5′-CTGCAGATGGAGCATGTTGT-3′ (sense) and 5′-TCTTCACGAGG AGGCTTGAT-3′ (anti-sense); Pdh, 5′-TGGTGGCATCCCGTAACTC-3′ (sense) and 5'(sense) and GTAACTC-T-p3'(sense); and GTand S18, 5′-TTTGCGAGTACTCAACACCAACA-3′ (sense) and 5′-CCTCTTGGTGAGGTCAATGTCTG-3′ (anti-sense).

Quantitative PCR was performed using the Power SYBR-Green PCR kit (4367659, Applied Biosystems, Foster City, CA, USA), according to the manufacturer’s guidelines. Relative transcript levels were calculated using the comparative Ct method (27), and the expression of S18 was used as an internal control.

### IgG Enzyme-Linked Immunosorbent Assay

Ninety-six-well plate pre-coated with 10-µg/mL goat anti-human Ig(H + L)-UNLB were washed with PBST (0.05% Tween 20 in PBS) and blocked with 1% BSA for 1 h. Then, the plates were washed and incubated for 1 h with the plasmablast culture supernatant and human reference serum serially diluted twofold from 250 ng/mL. The plates were then washed with PBST, incubated at room temperature for 1 h with goat anti-human Ig(H + L)-HRP diluted 1:5,000 in PBST, and developed by adding TMB substrate. The reaction was stopped with 2N sulfuric acid, and then, the absorbance was measured at 450 nm using a Sunrise microplate reader (Tecan, Männedorf, Switzerland).

### Transwell Migration Assay

To assess the chemotactic migration of plasmablasts toward CXCL12, *in vitro*-generated plasmablasts were harvested and washed twice with PBS. Then, 1 × 10^5^ cells were resuspended in 100 µL of the migration buffer (0.5% BSA-RPMI 1640) and added to the upper chamber of the transwell inserts (Transwell Permeable Support with a 5.0-µm polycarbonate membrane, 6.5-mm insert; 3421, Corning). Next, 600 µL of the migration buffer with or without 100-ng CXCL12 was added to the bottom chamber. After 2 h of incubation, the cells in the top chamber (i.e., non-migrated cells) were removed and those in the bottom chamber (i.e., migrated) were collected. Migrated cells were counted using a propidium iodide exclusion assay performed with an Accuri C6 flow cytometer.

### Intracellular Flow Cytometry

Plasmablasts were stained for intracellular Ki-67, phospho-MLC, and phospho-AKT according to the BD Phosflow Protocol III. Briefly, the cells were fixed in a pre-warmed BD Cytofix solution and permeabilized by incubation with chilled BD Perm Buffer III for 30 min. After permeabilization, the cells were washed three times with the flow cytometry buffer and incubated for 1 h with 0.25 µg of FITC mouse anti-human Ki-67. Then, the cells were washed and resuspended in 500 µL of the flow cytometry buffer. In total, 20,000 fixed cells were acquired and analyzed using an Accuri C6 flow cytometer.

### Western Blot Analysis

Protein extracts from plasmablasts were separated on 12% SDS-polyacrylamide gels and electrophoretically transferred to an Immun-Blot PVDF membrane for protein blotting (162-0177; Bio-Rad Laboratories). The membrane was blocked for 1 h with 5% BSA and then incubated overnight with Abs specific to the phospho-PDH-E1α subunit or PDH. Unbound primary Abs were removed by washing the membrane three times with TBS/0.1% Tween 20; this was followed by incubation with horseradish peroxidase-conjugated anti-rabbit or anti-mouse secondary Abs (diluted 1:3,000 in TBS/0.1% Tween 20). Proteins were visualized using SuperSignal West Pico Chemiluminescent Substrate and an ImageQuant LAS 4000 biomolecular imager (GE Healthcare Life Sciences). The ImageJ densitometry plug-in was used for quantitative analysis of Western blot images. Equal loading was confirmed by stripping the blot and re-probing for β-actin.

### Immunocytochemistry

To stain and assess the immunofluorescence of phosphorylated MLC, 2 × 10^5^ plasmablasts were harvested, washed with PBS, and seeded onto chamber slides. The cells were then fixed with 1% paraformaldehyde and permeabilized with 0.1% Triton X-100. Then, they were incubated for 1 h with anti-human pMLC Ab (diluted 1:100). After washing with PBS, the cells were incubated further for 1 h with a FITC-conjugated anti-rabbit secondary Ab (diluted 1:100 in PBS) and DAPI. Fluorescence was observed using an LSM 710 confocal microscope (Carl Zeiss AG, Oberkochen, Germany).

### Metabolite Analysis

Intracellular metabolite levels were measured by liquid chromatography (LC)–MS/MS analysis. Briefly, the cells were washed with PBS and H_2_O; this was followed by lysis with 80% cold methanol and vigorous vortexing. The lysates were harvested by centrifugation, and polar metabolites were retained as chloroform extracts in the aqueous phase. The aqueous phase was dried in a vacuum centrifuge, and the sample was reconstituted with 50 µL of 50% methanol. All standards, including surrogate internal standards, and solvents were purchased from Sigma-Aldrich or JT Baker (Phillipsburg, NJ, USA). The LC–MS/MS system was equipped with an Agilent 1290 HPLC (Agilent, Santa Clara, CA, USA) and a Trap 5500 (AB SciexLLC, Framingham, MA, USA). A Synergi fusion column (Synergi 4u-fusion RP 80A, 50 × 2.00 RP fusion Torrance, CA, USA) was used for separation.

### Measurement of Extracellular Flux

To immobilize the suspended cells, XF24 cell culture plate (100777-004; Seahorse Bioscience, North Billerica, MA, USA) was coated with Cell-Tak cell and tissue adhesive. Briefly, cultured plasmablasts were seeded on Cell-Tak-coated 24-well XF culture plate in XF media (non-buffered RPMI 1640 containing 10-mM glucose, 2-mM l-glutamine, and 1-mM sodium pyruvate; 102365-100; Seahorse Bioscience). Then, the extracellular flux rate was measured using a Seahorse Bioscience XF24 Analyzer according to the manufacturer’s protocol. The oxygen consumption rate (OCR) and extracellular acidification rate (ECAR) were measured under basal conditions after the addition of CXCL12. Inhibitors were added at the following final concentrations: oligomycin A, 1 µM; FCCP, 1.5 µM; rotenone, 0.1 µM; antimycin A, 1 µM; 2-DG, 10 mM; and DON, 5 µM.

### Metabolic Enzyme Activity Assay

Cellular PDH activity and lactate dehydrogenase (LDH) activity were measured in accordance with the instructions provided by the activity assay kits (MAK183 and MAK066, respectively; Sigma-Aldrich). Briefly, the cells were pretreated with AKT inhibitors and then stimulated with CXCL12 for 2 min at 37°C. The cells were extracted with assay buffer and clarified by centrifugation. Next, the extracts were incubated with the reaction mixture at 37°C for up to 30 min, and then, enzyme activity was measured spectrophotometrically and calculated *via* standard curve interpolation.

### Viral Transduction

The expression of PDH was knocked down using PDH-targeting MISSION RNA interference vectors, which were obtained through a partnership agreement between Sigma and the Mayo Clinic RNA Interference Technology Resource (TRCN0000028590 and TRCN0000028627). To improve viral transduction efficacy, we used the measles virus glycoprotein-displaying lentivirus transduction system (28). Lentiviruses that displayed measles virus glycoprotein and contained a small hairpin RNA (shRNA) complementary to PDH and an empty vector (pLKO.1) were generated by transient transfection of the vector together with plasmids psPAX2, pCG-Fdel24, and pCG-Hdel30 into the 293 T cell line. The supernatant containing the lentiviruses was collected, filtered through a 0.45-µm filter, and saturated by polyethylene glycol precipitation. The saturated virus was transduced into a GC-B cell differentiation system. To measure the transduction efficiency, PDH expression was analyzed by qPCR and Western blotting.

### Detection of Reactive Oxygen Species (ROS)

Cellular ROS were estimated in accordance with the instructions provided by the cellular (CM-H_2_DCFDA, C6827, Thermo-Fisher Scientific) and mitochondrial (MitoSOX Red Mitochondrial Superoxide Indicator, live-cell imaging, M36008, Thermo-Fisher Scientific) ROS assay kits. Briefly, cells were pretreated with these ROS indicators and then stimulated with CXCL12 for 15 min at 37°C. The cells were acquired and analyzed using an Accuri C6 flow cytometer.

### Institutional Review Board Approval

This study was approved by the institutional review board of the Asan Medical Center (approval number, 2013–0864). Informed consent was waived because there was no additional risk to the participants and their identities were anonymized and completely delinked from unique identifiers.

### Statistical Analysis

All experiments were repeated three or more times. Statistical significance was analyzed using *t*-tests and GraphPad Prism software (version 6). Data were expressed as the mean ± SD. A *p*-value of <0.05 was considered statistically significant.

## Results

### Generation of Plasmablasts Migrating Toward CXCL12 Using an *In Vitro* GC-B Cell Differentiation System

**Figure 1:**
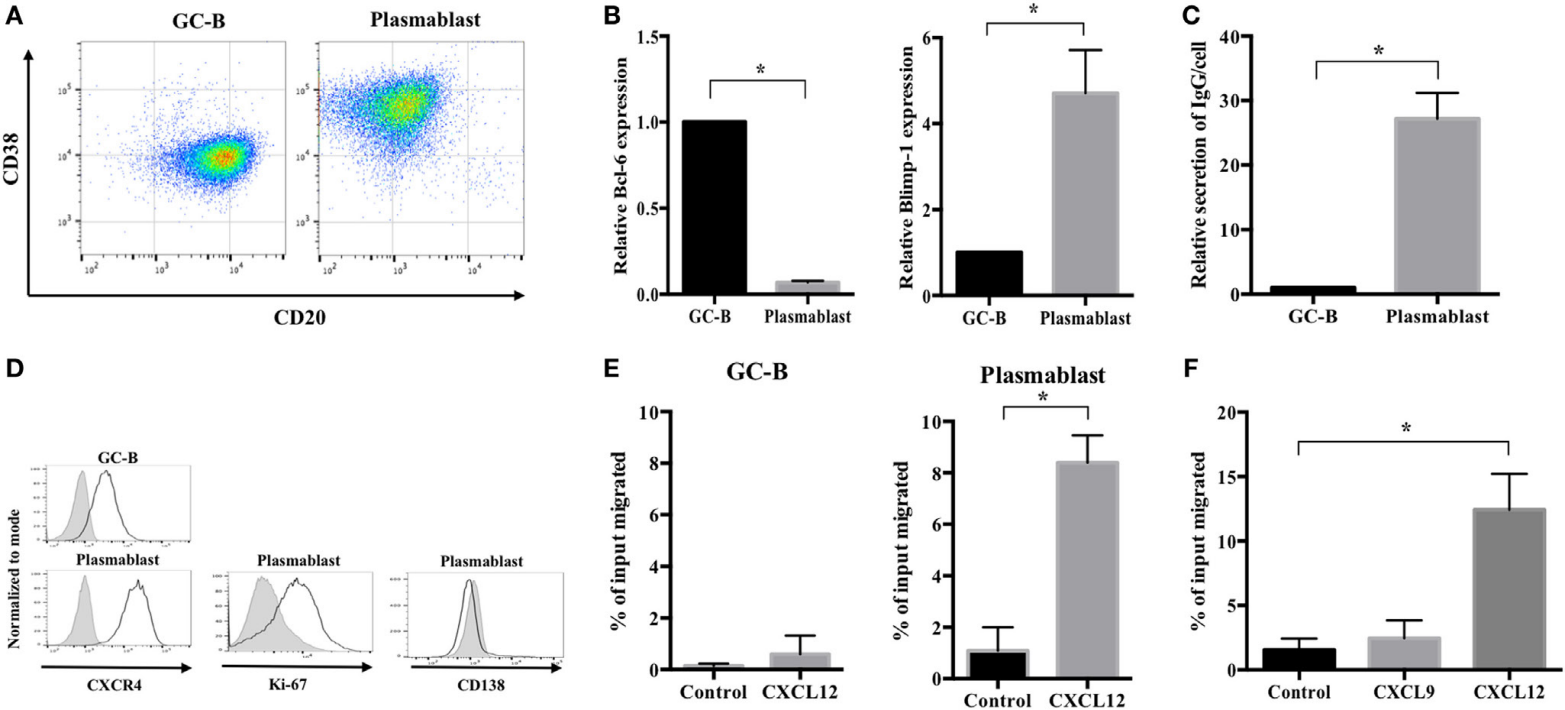
Generation of plasmablasts migrating toward CXCL12 using an *in vitro* germinal center-B (GC-B) cell differentiation system. Germinal center (GC) B cells were purified from human tonsillar mononuclear cells using MACS and cultured with IL-2 and IL-21 for 7 days. Cell differentiation markers, immunoglobulin (Ig) secretion, and migration properties were analyzed to assess the phenotype of migrating plasmablasts. **(A)** A representative image showing CD38 and CD20 expression of GC-B cells and cultured plasmablasts. **(B)** *Bcl-6* and *Blimp-1* expression levels in GC-B cells and plasmablasts were measured by quantitative PCR. **(C)** The amount of secreted Ig was measured by ELISA on culture Day 4 (GC-B) and Day 7 (plasmablast). The values of IgG secretion were normalized by the cell number of each sample. Plasmablasts showed a 27-fold increase in IgG secretion compared to GC-B cells. **(D)** Representative images showing the expression of CXCR4, Ki-67, and CD138 in GC-B cells and plasmablasts. **(E)** The CXCL12-induced migration of GC-B cells and plasmablasts. Plasmablasts showed a marked increase in migration toward CXCL12. **(F)** Specific migration of plasmablasts toward CXCL12 but not CXCL9. Data shown are means and SDs of three independent biological replicates. *p < 0.05 vs. GC-B or control.

It is extremely difficult to isolate human plasmablasts from peripheral blood because they are very scarce (29). Therefore, to study their migration, we first established an *in vitro* human plasmablast differentiation system using tonsillar GC-B cells. These cells were isolated by negative selection using MACS as previously described (30). The cells were then cultured for 4 days with IL-2 and IL-21 in the presence of CD40 ligand; this was followed by another 3 days of culture with IL-2 and IL-21 in the absence of CD40 ligand. GC-B cells differentiated into CD38^++^/CD20^−^ cells on Day 7 (Figure 1A). These cells showed a definite reduction in *Bcl-6* expression and marked induction of *Blimp-1* (Figure 1B). Furthermore, these cells showed significantly increased Ig secretion compared with GC-B cells (Figure 1C). These findings demonstrated evident differentiation into plasmacytoid cells. However, unlike terminally differentiated plasma cells, the cells differentiated from GC-B cells maintained proliferative capacity, as evidenced by Ki-67 expression and the lack of the terminal differentiation marker CD138 (Figure 1D). Importantly, the differentiated cells showed significantly greater migration toward CXCL12 (by 14-fold) and higher CXCR4 expression than GC-B cells (Figures 1D,E). Notably, plasmablast migration was CXCL12-specific as the plasmablasts barely moved toward CXCL9 (Figure 1F). These characteristics are consistent with those of plasmablasts migrating toward the bone marrow niche (31–33). Taken together, the findings show successful establishment of a human plasmablast development system that generates cells that specifically migrate toward CXCL12. This system enabled us to further examine the cellular metabolism that drives the migration of human plasmablasts to the bone marrow.

### CXCL12-Induced Migration Is Dependent on Glucose but Not on Glutamine

**Figure 2:**
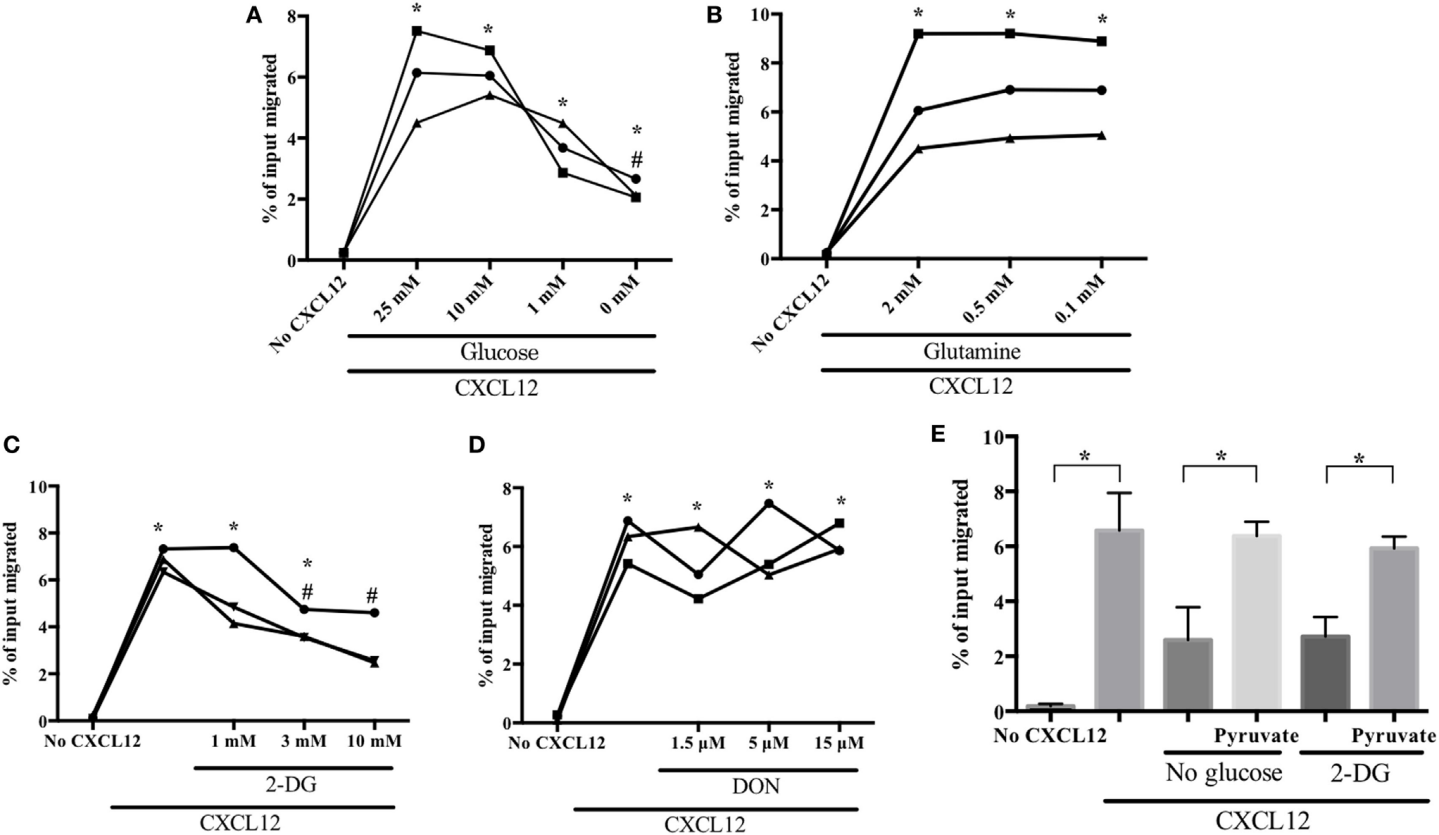
CXCL12-induced migration is dependent on glucose but not on glutamine. Plasmablasts were left to migrate in the absence or presence of CXCL12 for 2 h. Cells in the lower chamber of the transwell plate were collected and counted. Data are presented as the percentage of the total number of input cells that migrated. **(A)** CXCL12-induced migration is dependent on glucose concentration. Plasmablasts were cultured in decreasing concentrations of glucose and chemotaxis was assessed using the transwell migration assay. **(B)** Migration is not dependent on glutamine concentration. **(C,D)** Blocking glucose uptake led to a marked reduction in plasmablast migration, whereas blocking glutamine uptake had no effect. Cells were pretreated with the glucose analog 2-deoxyglucose (2-DG) or the glutamine analog 6-diazo-5-oxo-L-norleucine (DON), and then, CXCL12-mediated chemotaxis was estimated. **(E)** Pyruvate restored the decrease in CXCL12-induced migration brought upon by glucose deprivation and 2-DG treatment. Cells were left untreated or were treated with 1-mM methyl-pyruvate in medium containing 0-mM glucose or 2-DG. Each symbol in the graphs means biological replicates. Data shown are three independent experiments. *p < 0.05 vs. no CXCL12; ^#^*p* < 0.05 vs. CXCL12 with 25-mM glucose **(A)** or CXCL12 with 0 mM 2-DG **(C)**.

To identify the major metabolic pathways required for human plasmablast migration, we investigated the CXCL12-induced chemotaxis of plasmablasts in various concentrations of glucose and glutamine, which are the primary requirements of cellular metabolism (34, 35). We found that the decrease in the number of migrating cells was inversely proportional to the glucose concentration (Figure 2A). CXCL12 strongly induced plasmablast migration in the presence of 10-mM glucose; in comparison with this, migration decreased by 47% in the presence of 1-mM glucose and by 67% in the absence of glucose. Surprisingly, reducing glutamine concentration had no effect on the CXCL12-mediated migration of human plasmablasts (Figure 2B). To confirm these results, subsequent migration assays were performed in the presence of 2-deoxyglucose (2-DG; a glucose uptake blocker) and 6-diazo-5-oxo-L-norleucine (DON; a glutamine uptake inhibitor). Treatment with 2-DG led to marked inhibition of CXCL12-induced migration in a similar manner to glucose depletion, whereas DON treatment had no significant effect (Figures 2C,D). These results strongly suggest that the CXCL12-induced migration of human plasmablasts is dependent on glucose. To confirm the importance of glucose metabolism for plasmablast migration, we assessed whether pyruvate, which is catabolized from glucose, restored migration under glucose deprivation conditions. The results showed that pyruvate restored the decreased number of migrating cells under glucose deprivation conditions and upon 2-DG treatment (Figure 2E). Taken together, this indicates that the metabolic process underlying pyruvate generation plays a crucial role in the CXCL12-mediated migration of plasmablasts.

### CXCL12 Increases Aerobic Oxidation of Glucose for Migration

**Figure 3:**
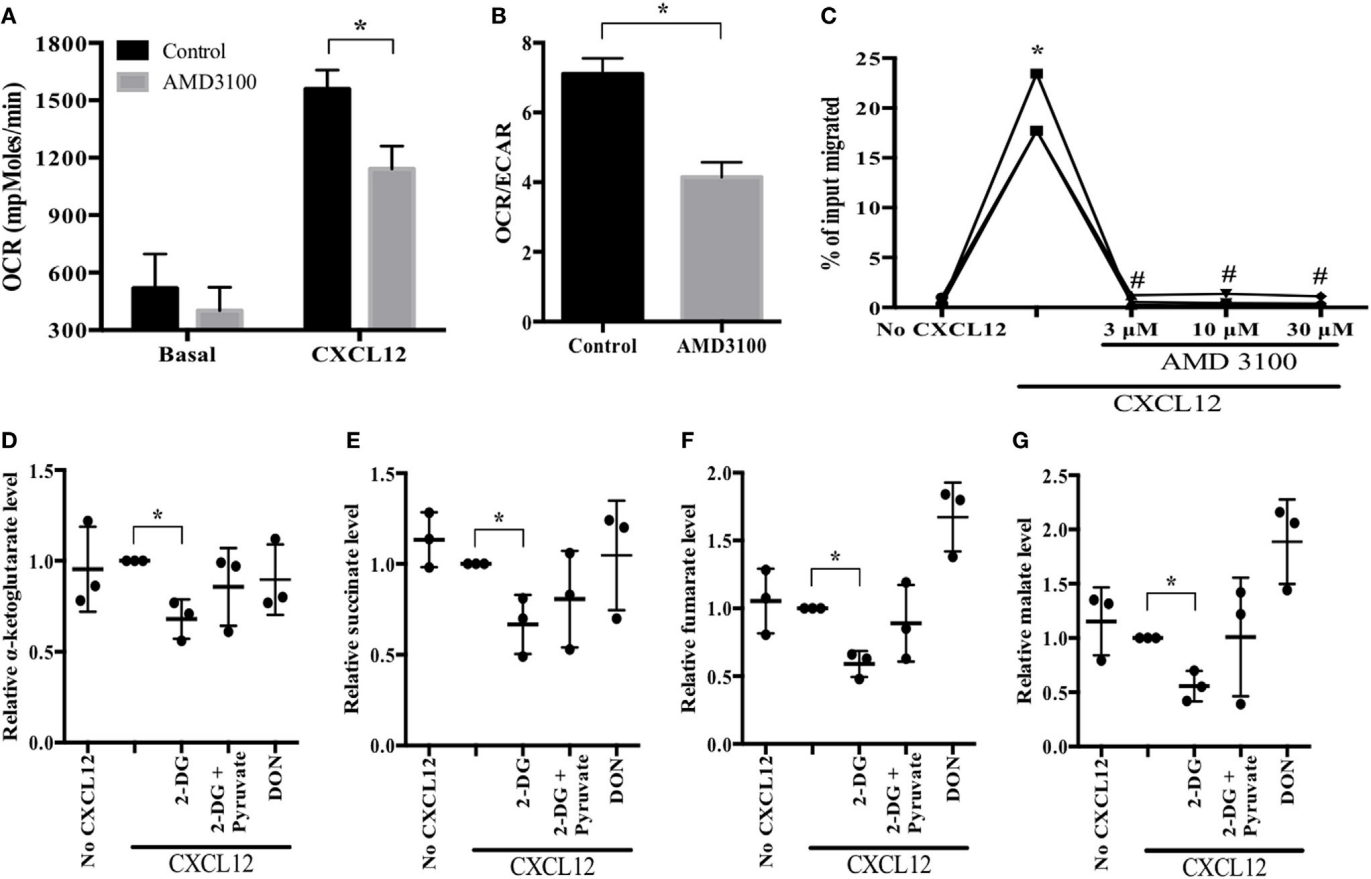
CXCL12 increases aerobic oxidation of glucose for migration. **(A,B)** Oxygen consumption rate (OCR) and the OCR/extracellular acidification rate (ECAR) ratio in the absence and presence of CXCL12. Cultured plasmablasts were seeded in Cell-Tak-coated 24-well XF plate, and then the extracellular flux rate was measured. CXCL12 increased OCR, and the CXCR4 antagonist AMD3100 inhibited the CXCL12-mediated OCR induction. **(C)** Migration in the presence of AMD3100. **(D–G)** Analysis of key metabolic intermediates of the tricarboxylic acid (TCA) cycle in plasmablasts. Plasmablasts were pretreated with CXCL12, 2-DG, pyruvate, and DON for 2 h. Then, polar metabolites from the cells were analyzed by liquid chromatography–mass spectrometry (MS)/MS. The bars indicate the relative levels of TCA cycle metabolic intermediates. 2-DG led to a significant reduction in the amounts of TCA cycle intermediates which were then restored in the presence of pyruvate. Data shown are results of three independent biological replicates. *p < 0.05 vs. control samples; ^#^*p* < 0.05 vs. CXCL12 in **(C)**.

To determine how glucose is metabolized during plasmablast migration toward CXCL12, we investigated metabolic parameters using an XF24 flux analyzer. CXCL12 stimulation increased OCR, whereas pretreatment with AMD3100, a CXCR4 antagonist, neutralized the CXCL12-mediated increase in OCR (Figure 3A; Figure S1 in Supplementary Material). AMD3100 also reduced the OCR/ECAR ratio induced by CXCL12 (Figure 3B). In addition, AMD3100 treatment led to a marked reduction in CXCL12-induced plasmablast migration (Figure 3C). Notably, CXCL12 stimulation augmented the accumulation of cellular and mitochondrial ROS (Figure S2 in Supplementary Material). These results confirm that CXCL12-induced migration is most likely dependent on glucose oxidation.

To corroborate the usage of glucose oxidation pathway in CXCL12-induced plasmablast migration, we compared the amount of TCA cycle metabolic intermediates in migrating plasmablasts (in the presence or absence of 2-DG) to confirm if pyruvate is utilized in the TCA cycle of migrating plasmablasts. 2-DG treatment of CXCL12-stimulated plasmablasts led to a marked reduction in the levels of all the tested TCA cycle intermediates; these levels were restored in the presence of pyruvate (Figures 3D–G). Conversely, DON treatment did not have a significant effect. Taken together, these results indicate that CXCL12 promotes glucose oxidation in the TCA cycle.

### CXCL12 Promotes PDH Activity in an AKT-Dependent Manner to Increase Plasmablast Migration

**Figure 4:**
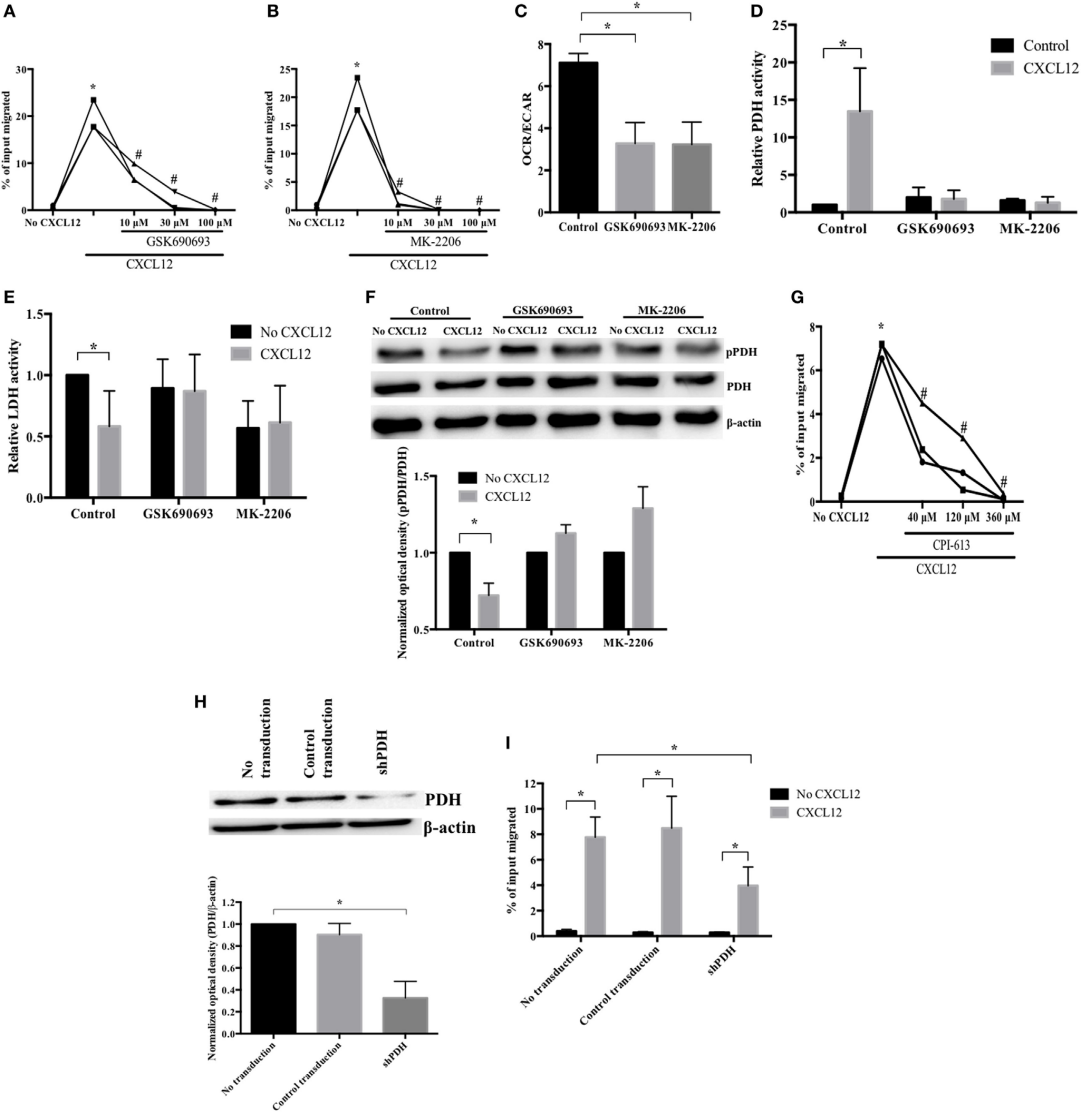
CXCL12 promotes pyruvate dehydrogenase (PDH) activity in an AKT-dependent manner to increase plasmablast migration. **(A,B)** AKT is essential for the CXCL12-induced migration of plasmablasts. Cells were pretreated with **(A)** GSK690693 or **(B)** MK-2206, and then, CXCL12-mediated chemotaxis was measured. **(C)** The CXCL12-mediated increase of oxygen consumption rate (OCR) is AKT-dependent. OCR/extracellular acidification rate was measured with AKT inhibitors (GSK690693 and MK-2206). **(D)** CXCL12 enhanced PDH activity in an AKT-dependent manner. Cells were left untreated or were treated with AKT inhibitors, and then, CXCL12-induced PDH activity was analyzed. **(E)** The inhibition of lactate dehydrogenase (LDH) activity by CXCL12 is AKT-dependent. **(F)** CXCL12 decreased the levels of phosphorylated PDH in an AKT-dependent manner. Cells were lysed, and then, protein extracts were analyzed by Western blotting with an anti-phospho-PDH-E1α antibody. The ImageJ densitometry plug-in was used for quantitative analysis. **(G)** PDH activity is required for CXCL12-induced migration. Plasmablasts were cultured with various concentrations of CPI-613, a PDH inhibitor, and then, chemotaxis was analyzed. **(H)** Knockdown of PDH successfully reduced PDH expression. Plasmablasts were transduced with vectors expressing small hairpin RNA complementary to PDH. Knockdown of PDH expression was verified by Western blot analysis. **(I)** PDH is essential for CXCL12-induced migration. Data shown are results of at least three independent biological replicates. *p < 0.05 vs. control samples; ^#^*p* < 0.05 vs. CXCL12 in **(B,C)**.

To examine the CXCL12-associated metabolic reprogramming involved in plasmablast migration, we conducted experiments using agents that block AKT pathways—the major drivers of plasmablast migration (23). As expected, treatment with the AKT inhibitors GSK690693 and MK-2206 (36) prompted a significant decrease in CXCL12-induced plasmablast migration (Figures 4A,B). In addition, the AKT inhibitors reduced CXCL12-induced OCR and the OCR/ECAR ratio (Figure 4C; Figure S1 in Supplementary Material). These results indicate that AKT is not only involved in the CXCL12-mediated signaling for migration but also in glucose metabolism, which is necessary for plasmablast migration.

For glucose to enter the TCA cycle, pyruvate must be converted into acetyl-CoA by PDH (37). When plasmablasts were exposed to CXCL12 for 5 min, PDH activity markedly increased by 13.5-fold (Figure 4D). Additionally, the activity of LDH, which catalyzes the conversion of pyruvate into lactate and favors anaerobic glycolysis, decreased (Figure 4E). AKT reduces the phosphorylation of the PDH-E1α subunit (38) as phosphorylation inhibits the activity of PDH, which promotes the conversion of pyruvate into acetyl-CoA (39). Therefore, we also tested whether the activity of PDH and LDH in migrating plasmablasts is AKT-dependent. Pretreatment with AKT inhibitors blocked the CXCL12-mediated increase in PDH activity and decrease in LDH activity (Figures 4D,E). These results show that CXCL12 modulates the activity of PDH and LDH to increase glucose conversion into acetyl-CoA and decrease glucose conversion into lactate in an AKT-dependent manner. Phosphorylation on the PDH-E1α subunit inhibits the PDH activity that promotes the conversion of pyruvate into acetyl-CoA (39). To check whether the CXCL12-mediated increase in PDH activity was due to a reduction in PDH-E1α phosphorylation, we estimated the levels of phosphorylated PDH-E1α by Western blotting. CXCL12 reduced the amount of phospho-PDH, which was then restored in samples treated with AKT inhibitors (Figure 4F). In addition, we performed a transwell migration assay with the PDH inhibitor CPI-613 and found that the decrease in CXCL12-induced migration was inversely proportional to CPI-613 concentration (Figure 4G). To further verify that PDH is essential for CXCL12-induced migration, we knocked down plasmablast PDH and performed another transwell migration assay. Transduction of the vector expressing shRNA complementary to PDH (shPDH) reduced PDH protein expression by 68% compared with control transduction (Figure 4H). The reduced expression of PDH resulted in a significant inhibition of the migration induced by CXCL12 (Figure 4I). These data indicate that PDH is required for the migration of plasmablasts toward CXCL12. Overall, these results show that CXCL12 increases the activity of PDH through AKT to induce plasmablast chemotaxis.

### Mitochondrial ATP Synthesis Is Essential for MLC Phosphorylation and Sustained AKT Activation

**Figure 5:**
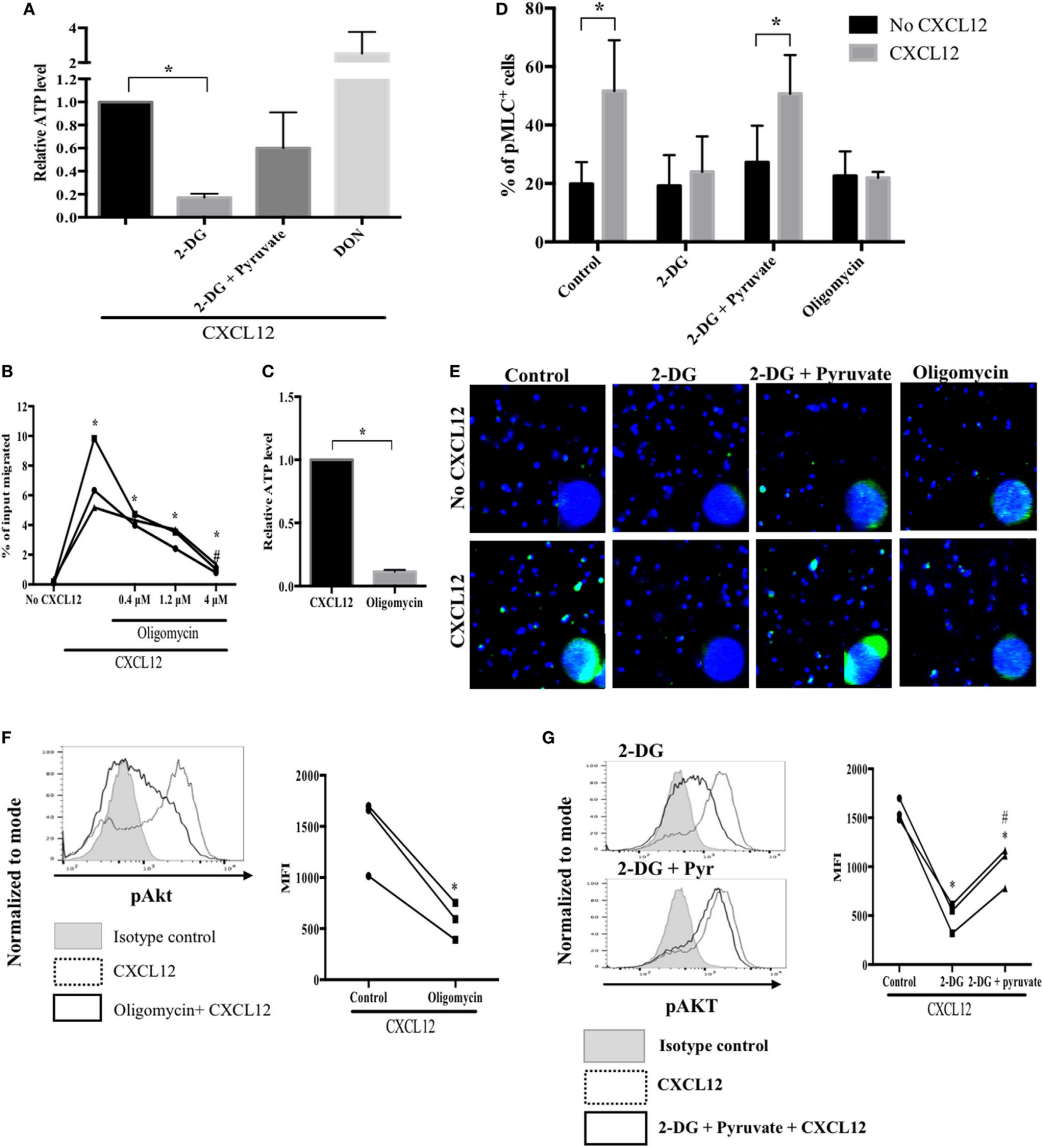
Mitochondrial ATP synthesis is essential for myosin light chain (MLC) phosphorylation and sustained AKT activation. **(A)** Treatment with a glucose analog, 2-DG, led to a marked reduction in cellular ATP levels. ATP levels in migrating plasmablasts were analyzed by liquid chromatography (LC)–MS/MS. **(B)** A mitochondrial ATP synthase inhibitor markedly suppressed CXCL12-induced migration. **(C)** Oligomycin reduced cellular ATP levels. **(D)** Mitochondrial ATP is required for MLC phosphorylation. MLC phosphorylation in plasmablasts was observed using an LSM 710 microscope. The number of cells in each field was counted, and the percentage of pMLC-positive cells was plotted. **(E)** Oligomycin reduced CXCL12-induced AKT activation. Cells were pretreated with oligomycin, and then, phospho-AKT levels were measured using flow cytometry. **(F)** 2-DG reversed the increased activation of AKT induced by CXCL12 while pyruvate recovered the AKT activation levels. Data shown are results of three independent experiments. *p < 0.05 vs. control samples; ^#^*p* < 0.05 vs. CXCL12 in **(B)** and 2-DG in **(G)**.

Blocking glucose metabolism greatly reduces cellular ATP levels and chemotaxis of migrating T cells (40). Therefore, to determine whether glucose is utilized during mitochondrial ATP production, we measured cellular ATP levels in migrating plasmablasts. Compared with the control, in plasmablasts, the exposure to 2-DG markedly decreased ATP levels (by 83%) and treatment with 2-DG together with pyruvate recovered ATP levels to 60% (Figure 5A). To examine whether mitochondrial ATP synthesis is important for plasmablast migration, we performed a transwell migration assay using oligomycin, a mitochondrial ATP synthase inhibitor (41). Oligomycin diminished both CXCL12-induced migration and intracellular ATP levels (Figures 5B,C). These results show that glucose is the primary driver of the mitochondrial ATP synthesis required for plasmablast migration. Next, we investigated the role of mitochondrial ATP in plasmablast migration. The phosphorylation of MLC modulates the activity of myosin II, thus promoting conformational changes that allow for actin–myosin interactions and triggering ATPase activity. Inhibiting myosin II ATPase activity adversely affects the polarity and motility of T cells (41, 42). CXCL12 was found to significantly increase the number of phospho-MLC-positive plasmablasts (Figures 5D,E), whereas treatment with 2-DG and oligomycin blocked the CXCL12-induced phosphorylation of MLC (Figures 5D,E). Furthermore, the reduced number of phospho-MLC-positive cells in the presence of 2-DG was reversed by pyruvate. Overall, these results suggest that mitochondrial ATP generation is essential for the phosphorylation of MLC, which is in turn necessary for cell migration.

Since AKT is a primary regulator of plasmablast migration, it is necessary to sustain the activation of AKT for continuous migration of plasmablasts; therefore, we examined if decreasing ATP levels by inhibiting glucose metabolism affects the maintenance of AKT activation. Oligomycin treatment reduced the CXCL12-mediated increase in AKT activation (Figure 5F). Furthermore, 2-DG markedly inhibited the phosphorylation of AKT induced by CXCL12. Notably, pyruvate restored the decreased levels of activated AKT under glucose deprivation conditions (Figure 5G). These results indicate that mitochondrial ATP is required for sustained AKT activation.

## Discussion

Following the germinal center reaction, developing plasmablast exploits two main chemotactic signaling to migrate: CXCL9, 10, and 11/CXCR3 axis, and CXCL12/CXCR4 axis (3). The former is crucial for migrating to inflammatory sites, whereas the latter is important for homing to the bone marrow, resulting in final differentiation to long-lived plasma cells and the provision of a steady level of protective Abs (31–33). Despite the importance of the migration of plasmablast to the bone marrow, the detailed metabolic mechanism remains to be elucidated, partly because of the lack of proper methods that provide enough number of migrating human plasmablasts. We have established a primary culture method that supports human GC-B cells to develop into plasmablasts that migrate only toward CXCL12 and not toward CXCL9. This unique system allows us to investigate the molecular mechanisms of metabolic pathways underlying human plasmablast migration to the bone marrow. Using this system, we found that plasmablast migration toward CXCL12 is highly dependent on glucose oxidation.

Since our study is, to our knowledge, the first investigation on the metabolic mechanism of plasmablast migration, we cannot compare our results directly with any previous study on plasmablasts. However, the results are in agreement with those of recent studies showing that glucose plays an important role in lymphocyte migration. Blocking glucose metabolism greatly reduces T cell migration (40). The migration of regulatory T cells requires glucose metabolism (43). Glucose was also essential in macrophage migration, which was confirmed by a marked reduction in the number of primary macrophages migrating toward CXCL12 in the presence of 2-DG (44). Unlike glucose, glutamine is reportedly not essential for cell migration, which is also consistent with our findings. Through the ^13^C-glutamine tracing approach, it was found that although glutamine is not required for cell migration, it fuels the proliferation of endothelial cells (45). However, there are few reports on the use of fatty acids in migration wherein fatty acid augmented the migration of breast cancer cells (46, 47). As plasmablasts effectively migrated toward CXCL12 in the transwell migration assay in the absence of a lipid component, fatty acids may not be essential for plasmablast migration.

Most cancer cells and activated lymphocytes exhibit the Warburg effect, implying that they do not use energy-efficient metabolism—even under oxygen-rich conditions—to enable mitochondria to generate the lipids, amino acids, and nucleosides necessary for rapid cell proliferation (48). Since proliferation is unnecessary during plasmablast migration, efficient production of ATP *via* glucose utilization in the TCA cycle is a reasonable metabolic mechanism for cell migration. A propensity of increased glycolysis in lymphocyte migration has been reported. The migration of regulatory T cells is dependent on the aerobic glycolysis (43). Blocking of T cell migration by 2-DG is associated with a dramatic reduction of the amount of cellular ATP (40). In our results, CXCL12 increased PDH activity, which is crucial for pyruvate to redirect toward the TCA cycle by conversion to acetyl-CoA. The CXCL12-mediated increase in PDH activity was directly confirmed by measuring its phosphorylation and activity levels. CXCL12 also increased oxygen consumption and ROS level. Moreover, 2-DG greatly reduced the migration, cellular ATP levels, and TCA cycle intermediates in plasmablasts. In addition, CXCL12 increased the oxygen consumption and mitochondrial ATP production in immature human blood cells (49). Therefore, CXCL12 is considered to play a key role in directing glucose to the TCA cycle by enhancing PDH activity to fuel plasmablast migration.

Glucose also can be metabolized to lactate, which does not consume oxygen (50, 51). LDH catalyzes the interconversion between pyruvate and lactate (52). Therefore, LDH may also have a role in glucose oxidation. In our results of the LDH activity assay, the activity was reduced in migrating plasmablasts after CXCL12 stimulation. The CXCL12-mediated decrease in LDH activity may have resulted from a decrease in the amount of available LDH substrate due to increased PDH activity. Moreover, LDH can also convert lactate back to pyruvate (53). Thus, further analysis is required to investigate the exact role of LDH in plasmablast migration to the bone marrow.

Previous studies have provided the evidence of altered immune function in patients with metabolic diseases, such as diabetes mellitus (54). Patients with diabetes have weak immune responses, including reduced Ig production, in response to infections (55). CXCR4-knockout mice exhibit impaired Ab responses and defective homing of plasma cells to the bone marrow (5, 6). In addition, about 25% of Waldenstrom macroglobulinemia cases have CXCR4 mutations and are resistant to ibrutinib treatment (56). Therefore, the present investigation may help future researches in the fields of humoral immunity and plasma cell-driven diseases, such as myeloma and autoimmune diseases.

Taken together, the results of the present study enabled us to elucidate the metabolic pathway underlying the migration of human plasmablasts as well as the signaling mechanism regulating this metabolic pathway. Glucose is essential for the migration of plasmablasts toward CXCL12. Glucose oxidation in the TCA cycle is facilitated by an AKT-dependent increase in PDH activity mediated by CXCL12. To our knowledge, this study is the first to investigate the metabolic mechanisms underlying plasmablast migration toward CXCL12 in humans; therefore, our findings can form the basis for further studies on the metabolic control of humoral immunity and plasma cell-related diseases, such as myelomas and autoimmune diseases.

## Ethics Statement

This study was approved by the institutional review board of the Asan Medical Center (approval number, 2013–0864). Informed consent was waived because there was no additional risk to the participants and their identities were anonymized and completely delinked from unique identifiers.

## Author Contributions

C-SP and H-KP conceived and performed experiments. H-KP, BN, YL, and Y-WK carried out the experiments. Y-SC and JC prepared the samples. C-SP, H-KP, JR, and JS interpreted the results. H-KP wrote the manuscript with support from C-SP. C-SP supervised the project. All authors contributed to the final manuscript.

## Conflict of Interest Statement

The authors declare that the research was conducted in the absence of any commercial or financial relationships that could be construed as a potential conflict of interest. The reviewer FL and handling Editor declared their shared affiliation.

## Supplementary Materials

Figure S1 CXCL12 increases oxygen consumption rate (OCR) in an AKT-dependent manner. Cultured plasmablasts were pretreated with AMD3100, GSK690693, and MK-2206 for 2 h. Then the extracellular flux rate was measured. CXCL12 increased both the OCR and the extracellular acidification rate (ECAR); however, the AKT inhibitors GSK690693 and MK-2206 reduced the OCR but not the ECAR. The data are representative of three independent experiments.

Figure S2 CXCL12 augments mitochondrial reactive oxygen species (ROS) accumulation. **(A)** CXCL12 stimulation accompanies cellular ROS accumulation. Plasmablasts were pretreated with CM-H_2_DCFDA and then by CXCL12. The amount of cellular ROS was confirmed by measuring the level of DCF using flow cytometry. **(B)** CXCL12 induces mitochondrial ROS accumulation. Plasmablasts were pretreated with MitoSOX red reagent, and then, mitochondrial ROS levels were measured by flow cytometry. Data are representative of two independent experiments.
