# Supplementary figures and images for: Human Plasmablast Migration Toward CXCL12 Requires Glucose Oxidation by Enhanced Pyruvate Dehydrogenase Activity via AKT
Source: Front Immunol. 2018 Jul 27;9:1742. doi: 10.3389/fimmu.2018.01742 (PMC6072847; doi:10.3389/fimmu.2018.01742)

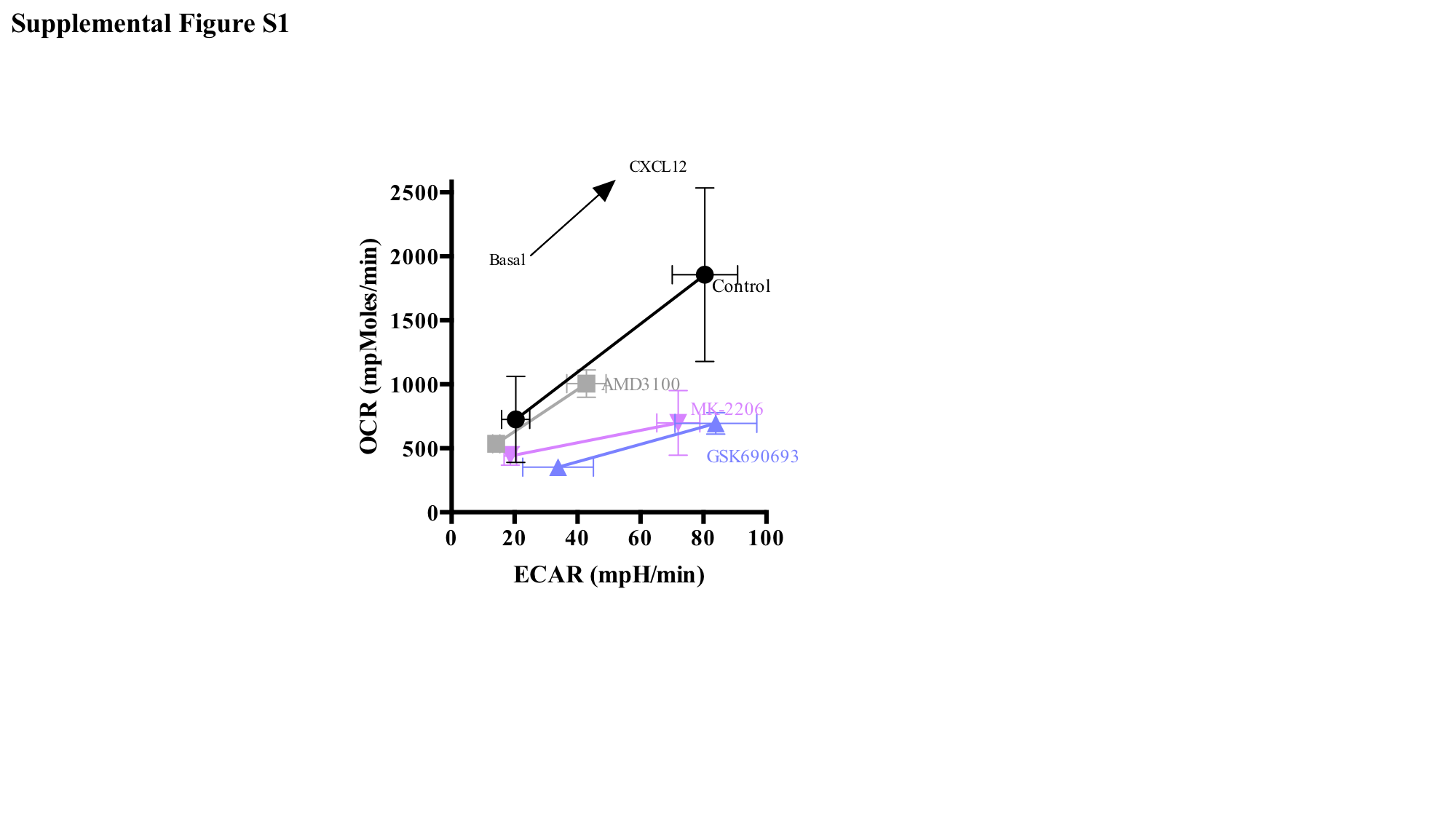

Supplement: Figure S1 — CXCL12 increases oxygen consumption rate (OCR) in an AKT-dependent manner. Cultured plasmablasts were pretreated with AMD3100, GSK690693, and MK-2206 for 2 h. Then the extracellular flux rate was measured. CXCL12 increased both the OCR and the extracellular acidification rate (ECAR); however, the AKT inhibitors GSK690693 and MK-2206 reduced the OCR but not the ECAR. The data are representative of three independent experiments. [file image_1.tiff]

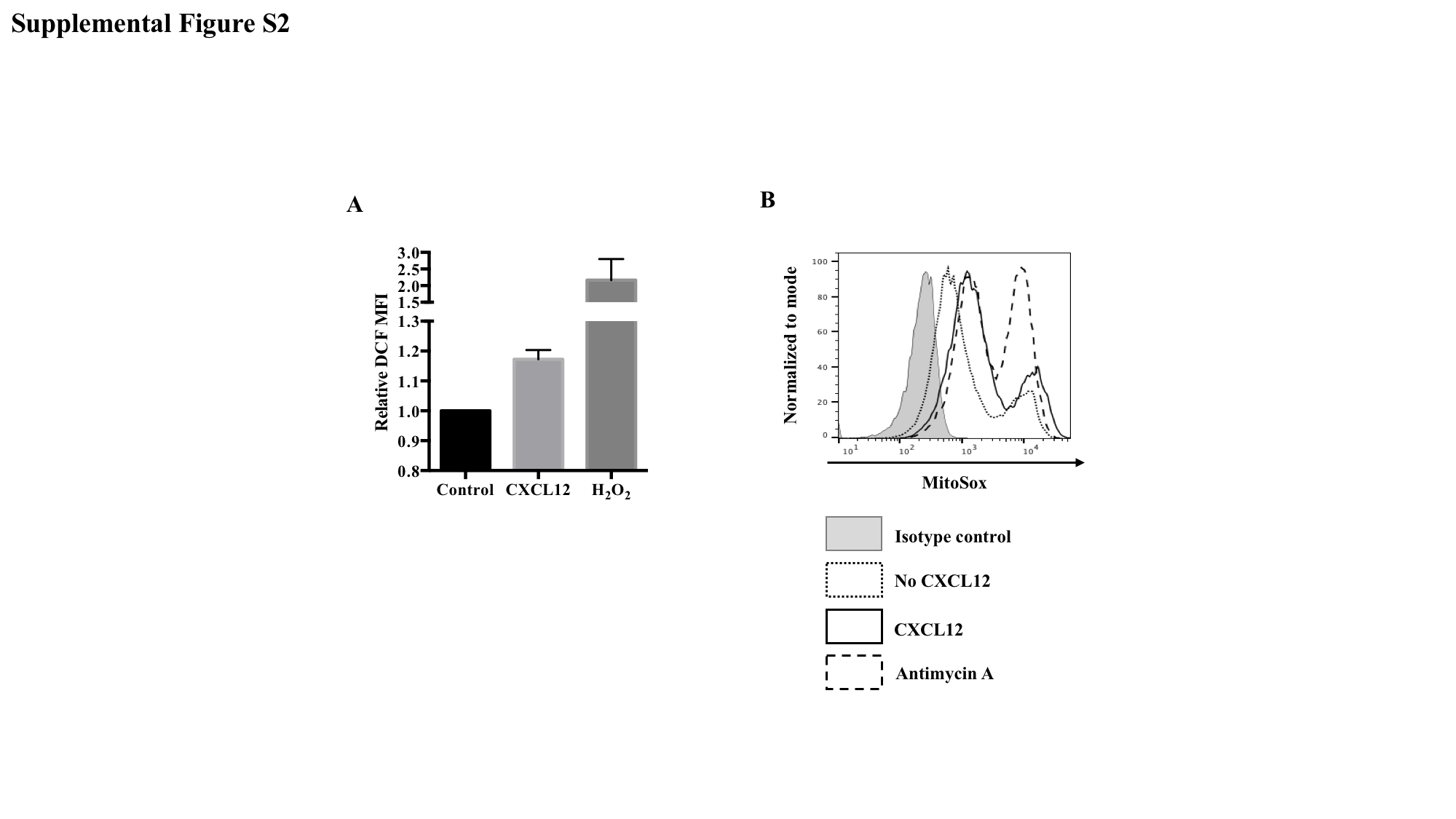

Supplement: Figure S2 — CXCL12 augments mitochondrial reactive oxygen species (ROS) accumulation. (A) CXCL12 stimulation accompanies cellular ROS accumulation. Plasmablasts were pretreated with CM-H2DCFDA and then by CXCL12. The amount of cellular ROS was confirmed by measuring the level of DCF using flow cytometry. (B) CXCL12 induces mitochondrial ROS accumulation. Plasmablasts were pretreated with MitoSOX red reagent, and then, mitochondrial ROS levels were measured by flow cytometry. Data are representative of two independent experiments. [file image_2.tiff]
